# Initial indication of treatment in 60 patients with sleep obstructive ventilatory disturbance

**DOI:** 10.1016/S1808-8694(15)31242-8

**Published:** 2015-10-20

**Authors:** Paulo de Tarso Moura Borges, Jorge Rizzato Paschoal

**Affiliations:** Master in Medicine, UNICAMP, Assistant Professor, Discipline of Otorhinolaryngology, Federal University of Piauí; Ph.D. in Otorhinolaryngology, UNICAMP, Assistant Professor, Discipline of Otorhinolaryngology, UNICAMP. Medical School, Universidade Estadual de Campinas (UNICAMP)

**Keywords:** apnea, sleep apnea syndromes, sleep apnea, obstructive

## Abstract

**Aim:**

The author present a retrospective descriptive study of 60 patients with sleep obstructive ventilatory disturbance who have taken medical advice at the Centro Campinas de Otorrinolaringologia e Cirurgia de Cabeça e Pescoço during a period of three years. All the patients have been examined after standardized protocol and decisions related to the treatment have been taken after systematic multidisciplinary discussion.

**Study design:**

clinical retrospective.

**Material and method:**

The patients were distributed into two groups according to the proposal of surgical and non-surgical treatment. After so, they were studied according to the model of treatment proposed and the main propaedeutic findings: respiratory disturbance index (RDI), body mass index (BMI), cephalometric analysis and Müller maneuver. The main features were compared - isolated or in association - with the model of treatment proposed.

**Conclusion:**

Amongst several conclusions obtained, the most important were: surgical and non-surgical treatment were indicated almost in the same proportion for of snoring; surgical treatments were most indicated for snoring and Apnoea-Hipopnoea Syndrome, despite of its modality; RDI, BMI and cephalometric analysis and Müller maneuver had no influence at any therapeutic modality; the therapeutic decision was taken after standardized protocol and systematic multidisciplinary discussion, where each case was discussed individually.

## INTRODUCTION

Diagnosis and therapeutic management of sleep obstructive disorders (SOD) have been presented in the literature as a challenge resulting from multifactorial pathophysiology^1^. The spectrum of Obstructive Sleep Apnea/Hypoapnea syndrome (OSAHS) may range from simple snoring to interference in social situations and severe situations in which apnea may lead to death^2^. OSAHS affects 4–7% of the general adult population^3^. Owing to its prevalence, it is currently considered a major public health concern, with severe physical and social consequences if not properly treated^3^^,^^4^.

These disorders affect mainly middle-aged patients that are professionally active and may generate high losses and absences from work^5^. Medical costs of SOD may be significantly reduced when effective diagnosis and treatment are performed early^6^.

The different modalities of treatment proposed for snoring and OSAHS may involve participation of professionals from different areas^7^ and should be adapted to the individual characteristics of patients and the nature of the obstruction. The option for many different treatments depends on the history of the patients, morbidity of the process and respective side effects and benefits of these therapeutic procedures^8^.

In addition to the multifactorial origin of the sleep obstructive disorders that many times hinders the precise diagnosis of the factors related with obstruction and its repair, most patients have difficult to comply with treatment and follow-up. Given that most of these patients are obese and many abuse alcoholic drinks, eat before going to bed and can only sleep in dorsal decubitus, the main difficulty lies in changing life style, preventing the worsening of these aggravating factors. Many start treatment but give it up right after.

Many patients do not accept surgical treatment, especially the most complex procedures. But even such treatment options are effective only if associated with behavioral treatment.

The purpose of the present study was to identify the influence of polysomnography in body mass index, cephalometry and Müller maneuver in initial indication of treatment in the group of patients that have sleep obstructive disorders and to review the most indicated procedures.

## MATERIAL AND METHODS

We retrospectively studied the data obtained in the medical charts of 60 patients of both genders in the age range of 19 to 70 years. Patients were originated from a sequential and non-selected sample that came for otorhinolaryngological treatment at Centro Campinas for Otorhinolaryngology and Head and Neck Surgery, with complaints of snoring, excessive daily drowsiness or non-relaxing sleep, between January 1998 and December 2001.

The charts of patients that had those complaints but also manifested craniofacial deformities or were holders of rhinosinusal and/or pharyngeal diseases, such as tumors, polyps and cysts, were excluded from the study.

The study was performed independently from gender and age range.

All patients had been submitted to clinical otorhinolaryngological examination according to a standardized protocol, which comprised anamnesis and physical examination.

Body mass index (BMI) was calculated for all patients. BMI is the correlation between weight of the subjects in kg and height in square meters. We considered to be obese patients that had BMI > 27.3.

The complementary tests we used were:

1. Videofibronasopharyngolaryngoscopy, comprising static and dynamic upper airway assessment, from the nasal cavity to the larynx, with investigation of Müller maneuver. This maneuver was performed for both rhinopharynx and oropharynx/hypopharynx to observe the occurrence of narrowing in these regions. It was considered positive when there was narrowing and negative when there wasn't.
2. Whole-nigh polysomnography, according to the standardization;
3. Teleradiography in profile, with cephalometric analysis, considering the following linear measurements in the assessment of upper airways: posterior air space (PAS), defined as space behind the tongue and limited by soft tissues; distance from the posterior nasal spine to posterior margin of the soft palate (PNS-P), which determined the length of the soft palate; distance from the mandibular plan to hyoid bone (MP-H)^9^^,^^10^. We used as comparative parameters the values considered to be normal by Riley et al.^9^: PAS 3 11 ± 1mm, Mp-H £ 15.4 ± 3mm and PNS-P £ 37 ± 3mm, even though they were subject to criticism resulting from absence of cephalometric Brazilian standard, considering the complexity of our racial composition.

Medical charts were divided into four groups, according to the classification into snoring, mild, moderate or severe OSAHS^11^:

Group 1: patients with snoring;
Group 2: patients with mild OSAHS;
Group 3: patients with moderate OSAHS;
Group 4: patients with severe OSAHS.

The differentiation between patients with snoring and OSAHS was made by the individual polysomnographic assessment, based on apnea and hypoapnea index (IAH), which adds up the number of respiratory events per hour of sleep.

Patients with IAH < 5 events per hour of sleep were considered as those who had snoring condition, patients with IAH between 5 and 15 per hour of sleep had mild OSAHS; IAH above 15 and below 30 comprised patients with moderate OSAHS, and those with IAH above 30 events were those with severe OSAHS 10.

Patients were divided into 2 subgroups concerning indication of treatment: non-surgical and surgical treatment. Non-surgical treatment included: behavioral changes, intraoral devices (AIO), drug treatment and CPAP. Behavioral changes in our study included weight loss, no intake of alcohol and change in sleeping position. Drug treatments used were tricyclic antidepressant and aminophylin.

As to surgical treatment, we indicated uvulopalatopharyngoplasty (UPFP), UPFP associated with AIO, laser-assisted uvulopatoplasty (LAUP), LAUP associated with AIO, LAUP associated with septoplasty, LAUP associated with septoplasty and turbinectomy, mandible advancement associated with UPFP, septoplasty associated with uvulectomy, tracheostomy, uvulectomy, hyoid elevation and tonsillectomy.

We studied the initial treatment modality indicated in each subgroup concerning respiratory disorder (snoring, mild, moderate or severe OSAHS), BMI (< 27.3 and ^3^ 27.3), cephalometric measurements (PAS, PNS-P and Mp-H) and Müller maneuver positive in rhinopharynx and/or oropharynx/hypopharynx. We only considered the medical charts of patients that had been submitted to all those exams in the studied period.

It was a descriptive study.

The project was approved by the Research Ethics Committee, Medical School, UNICAMP.

## RESULTS

The group with indication of non-surgical treatment comprised medical charts of 19 patients distributed according to the disorder into: 5 patients with snoring, 7 with mild OSAHS, 2 with moderate OSAHS and 5 with severe OSAHS.

The most indicated non-surgical treatment in cases of snoring was guidance on behavioral changes, in 3 (60%) cases. For patients with mild OSAHS they indicated AIO in 4 cases (57.14%). For those with moderate OSAHS, AIO and drug treatment were the most indicated procedures, similar in both groups. In severe cases of OSAHS, the most frequently indicated treatment was CPAP, with 2 indications (40%).

The group with surgical indication had 41 patients distributed according to the criteria into 6 patients with snoring, 13 with mild OSAHS, 6 with moderate and 16 cases with severe OSAHS.

Uvulectomy was the most frequently indicated surgery for snoring, with 3 indications (50%). In the groups with mild, moderate or severe OSAHS, UPFP was the most indicated procedure, with 9 (69.23%), 3 (50%) and 11 (68.75%) indications, respectively.

As to the most indicated treatment approach comprising BMI, AIO was the non-surgical procedure most predominantly recommended in patients with BMI values below 27.3 (5 patients = 55.55%). Behavioral change was the most common treatment in patients with values equal or better than 27.3 (4 patients = 40%).

UPFP was the most indicated surgical procedure both in patients with BMI < 27.3 (14 patients = 56%) and patients with BMI ^3^ 27.3 (11 patients = 44%). As to cephalometric measurements, behavioral changes were the most frequent non-surgical procedure to be indicated with PAS < 11 (7 patients = 43.74%). The patients were divided as follows: 3 snoring conditions, 3 mild OSAHS and 1 severe OSAHS. Three of them presented BMI < 27.3, 2 with snoring and 1 with mild OSAHS. The others were obese patients and they were divided as follows: 1 with snoring, 2 with mild OSAHS and 1 with severe OSAHS. AIO was the most frequent indication in patients with PAS ^3^ 11 (2 patients = 66.67%). One of the patients with mild OSAHS was obese and the other, with snoring condition, presented BMI = 24. All patients presented PNS-P^3^ 37 and only two patients presented Mp-H < 15.4, that is, practically all of the cephalometric values were above the normal standards in the literature. Therefore, these measurements were not considered in our study.

UPFP was the most frequently indicated surgery when we considered PAS, regardless of its variation: 13 patients with PAS < 11 (31.71%) and 12 patients with PAS ^3^ 11 (29.27%).

The 12 patients with PAS ^3^ 11 were distributed as 2 cases of snoring with 1 obese subject, 5 cases of mild OSAHS, with 1 obese subject, and 5 cases of severe OSAHS with 3 obese subjects.

## DISCUSSION

Multifactorial pathophysiology in sleep obstructive disorders is a continuous challenge to treatment 1. These disorders comprise snoring and OSAHS, and their different modalities. Treatment is based on anatomical and clinical affections, severity of diseases and risk factors^7^^,^^12^^,^^13^. The experience of the specialist in sleep disorder is essential for the success in the resolution of these problems. All therapeutic options can be discussed with the patient, focusing on benefits and possible failure^14^. The preference of patients for therapeutic options has a relevant role in the process. The treatment should always involve the participation of professionals from different areas^7^.

Given that the physical examination is not sensitive nor specific^15^, in our service we follow a routine that comprises directed anamnesis, videofibronasopharyngolaryngoscopy, cephalometrics and polysomnography. All exams are studied by a multidisciplinary team that comprises otorhinolaryngologists, neurologists, buccomaxillo-facial surgeons and speech and hearing therapists, and the decision-making is a joint process.

Müller maneuver is performed when the patient is awake. Even though it tries to simulate what happens during sleep, it may result in mistaken interpretations, given that the patient that is awaken does not relax the central command he/she has^16^.

Cephalometric studies vary according to ethnics. In our country, owing to major racial variation, failures in interpretation may be more severe.

The result of both studies, even though limited because the patient is awake, contributes to diagnostic improvement^4^.

Treatment success many times implies drastic modifications to lifestyle, compliance of the patient is difficult to be obtained in such cases. Reduction of body weight, interruption of drugs as benzodiazepine, no alcoholic drinks and change of sleeping in dorsal decubitus are some examples^12^^,^^14^. As to obesity, it is questioned whether it may be the cause or consequence of sleep disorders^17^.

The use of intraoral devices (AIO) may be uncomfortable because it causes muscle pain or pain in the temporomandibular joint^8^.

CPAP may not be properly used as a result of the side effects^8^^,^^12^.

The results of surgical procedures are many times of questionable value. They may promote initial improvement, with recurrence of later symptoms^18^^,^^19^. Other patients may not manifest any improvement.

The most frequently performed surgical procedure was UPFP. However, no diagnostic method is capable of predicting correctly its success, being considered effective only in 50% of the cases^18^^,^20., 21., 22..

In our sample, there was predominance of surgical treatment, in the proportion of 2:1. In fact, most of the patients in the sample had some indication of surgical approach.

The indication of surgical treatment compared to non-surgical treatment had the same proportion in cases of snoring. None of the 11 patients with snoring was obese and PAS was reduced in most of them (8 patients), confirming the concept that the pharynx is the main anatomical site of obstruction. The process of deobstructing the region, however, does not necessarily have to be surgical in snoring.

In OSAHS, surgical indication was predominant in 3 modalities (mild OSAHS = 2:1; moderate = 3:1; and severe = 3:1). Pinto & Colombini^23^ reported that patients with moderate and severe OSAHS, with good general health status, should be preferably submitted to surgical treatment. According to Piccirillo et al.^13^, the surgery should be the preferred treatment for snoring patients and those with mild OSAHS. In cases of moderate and severe OSAHS, it is indicated only in patients that refuse CPAP. It is worth pointing out that IDR > 30 is considered poor prognosis for most surgeries^24^. The surgical treatment, however, is not necessarily the definite treatment. Many times it will be complemented with other clinical interventions, if not surgical.

The most frequent surgical treatment indicated was UPFP, especially in OSAHS, regardless of severity. In the literature, we found reference to the best results of UPFP in patients with mild and moderate OSAHS, even though only surgery may not be curative25., 26., 27.. IDR < 30 is a predictive positive factor for better results in UPFP^28^.

In snoring, there was prevalence of surgical indications that involved the uvula and/or adjacent soft palate^4^^,^^26^^,^^29^.

There was predominance of surgical indication that implied direct work with redundant soft tissues (UPFP, LAUP, uvulectomy), compared to the indirect work and aiming at opening respiratory space (tracheostomy, hyoid elevation). Currently, the surgical treatment proposed tends to be a complementary procedure according to the case needs^30^.

Nasal obstructive factors did not play an important role in the surgical indication – 3 combined septoplasties. Similarly, the skeletal configuration did not play an important role - one indication of mandibular advancement, one of hyoid elevation, and one of tracheostomy. Isolated nasal surgery is of limited use and may be performed as a complement to pharyngoplasty or to reduce the pressure and improve tolerance to CPAP31., 32., 33.. Mandibular advancement surgery is indicated in retrognathism and hyoid elevation brings forward the tongue without mandible movement^34^. Tracheostomy is indicated in severe OSAHS with impairment of the general health and when the patient does not respond to other less aggressive procedures^4^^,^^13^^,^^14^^,^^35^^,^^36^.

Non-surgical treatments that were the most indicated where behavioral changes and AIO.

Weight loss is considered of extreme importance in patients with high BMI, which may reduce or event cure OSAHS. It should be encouraged in all obese patients^12^^,^^14^^,^^29^^,^^37^. No abuse of alcohol also prevents exacerbation of OSAHS 4 and dorsal decubitus worsens it^12^. Some patients present apnea only in this position^38^.

In patients with primary snoring and mild OSAHS that did not respond or did not have indication for behavioral changes, the use of AIO is indicated^24^. It may also be indicated in moderate and severe OSAHS, in patients that refused treatment with CPAP or that could not be operated on 39. In two patients, clinical conditions did not enable, permanently or temporarily, surgery under general anesthesia. CPAP was indicated. The use of CPAP is an effective treatment but we should bear in mind that once the disorder is not cured, it should be used for the entire life of the patient^13^^,^^14^^,^^18^^,^^40^.

In 2 of them we found subclinical neurological affections, expressed as mixed apnea, which made us decide for drug treatment.

BMI did not influence the non-surgical treatment, both in modality and in correlation with the disorder presented by the patients.

We found greater proportion of obese patients with severe OSAHS when we compared them to non-obese patients (2.5:1). Even among them, there was a predominance of UPFP. In general, UPFP is the most indicated surgical procedure. However, postoperative weight gain may hinder the initial result^26^.

Even though the study by Doghramji et al^22^ indicated that cephalometric radiography and Müller maneuver can not be used reliably to predict the surgical success of UPFP, to present these two methods seem to be the most useful ones^27^. Many authors consider that in OSAHS there is diffuse impairment of the airways, more than in a localized process. No method would be able to correctly predict the success of the surgical correction^18^^,^^41^^,^^42^. According to Metes et al.^42^, even if the retropalatal region is identified as the site of obstruction, the location does not predict the success of UPFP.

Millman et al.^28^ performed a study to determine whether polysomnography, cephalometry and anthropometric data could predict the success or failure of UPFP. A retrospective study with 46 patients submitted to this surgery was performed and the authors concluded that IDR < 38 and Mp-H £ 20mm, in addition to absence of retrognathia, were predictive factors of surgical success.

Measurements of PNS-P and MP-H are predominantly above the mean reported by the literature (93.3% and 96.7% of the cases, respectively). Even though the comparison is made with data from other racial groups, they are strongly suggestive of the reduction of oropharyngeal residual area and consequent reduction of air column permeability, strengthening the idea of indication of predominant surgical treatment. They are findings comparable to those described by FARIA^43^, in his master dissertation.

Similarly, there was predominance of the indication of surgical treatment regardless of the values PAS < 11 or PAS ^3^ 11. As to PAS < 11, we found surgical indication compared to non-surgical proportion of 1.6:1. In cases of PAS ^3^ 11, the same proportion was 5:1.

When we compare the type of ventilation disorder and PAS, we observe higher occurrence of mild and severe OSAHS in patients with PAS < 11 (13 and 14 patients, respectively, out of a total of 42). The others were divided into 8 patients with snoring and seven with moderate OSAHS. When both variables (ventilation disorder and PAS < 11) are compared with BMI, we found 20 non-obese patients and 22 obese patients. BMI had practically no importance in type of ventilation disorder as an indication of surgical treatment. Considering that these patients had indication of surgical treatment versus non-surgical treatment in the proportion of 1.6:1, we have the impression that none of the studied variables had a preponderant role in the suggested treatment modality.

Patients with PAS ^3^ 11 were distributed as 3 snoring patients, 7 with mild OSAHS, 1 with moderate and 7 with severe OSAHS. When we compared BMI, 11 were non-obese and 7 were obese. Given that in these cases there was predominance of surgical treatment, whose most frequent modality was UPFP, it seems that the volume of the palatine tonsil is the most important aspect in the decision-making for this therapeutic modality. The mass effect, resulting from this hypertrophied organ is responsible for the paradoxically increased value of PAS, given the occurrence of a significant obstructive phenomenon.

We found predominance of positive Müller maneuver at 2 levels (23 patients) in relation to negative result in both (7 patients), defining a proportion of 3.3:1.

Among the patients who had positive Müller maneuver in 2 levels, we found 4 with snoring, 8 with mild, 2 with moderate and 9 with severe OSAHS. As to BMI, 12 were obese and 11 were non-obese. We found 15 patients with PAS < 11 and 8 with PAS ^3^ 11, in the proportion of 1.4:1. In these patients, we indicated surgery in 16 patients, and UPFP was the most indicated one in 9 cases. Seven patients had indication of non-surgical treatment, in which there was predominance of AIO indication (4 patients). Two had indication of CPAP.

The positive Müller maneuver in 2 anatomical sites does not seem to have correlation with the nature of the obstructive process nor with BMI. Conversely, it seems to reinforce the need to expand the airways in the rhinopharynx and ororpharynx/hypopharynx, be it surgically or through use of AIO as the first measure.

Among the negative results in 2 levels, we found 1 case of snoring, 3 with mild, 1 with moderate and 2 cases with severe OSAHS. As to BMI, 3 were obese and 4 were non-obese. We found 5 patients with PAS < 11 and 2 with PAS ^3^ 11. In these patients, we indicated surgery in 6, 3 UPFP and 3 uvulectomy.

Despite the negative result in Müller maneuver in two sites, we were faced with indications that were aimed at expanding the space available in the rhinopharynx and oropharynx/hypopharynx through 2 types of surgeries that were the most indicated.

Among the 23 patients with positive result for Müller maneuver only in the rhinopharynx, we found 4 with snoring, 7 with mild, 4 with moderate and 8 with severe OSAHS. As to BMI, there were 11 obese ad 12 non-obese subjects. We found 18 patients with PAS < 11 and 5 with PAS ^3^ 11. In these patients, surgery was indicated in 13, and 10 were UPFP. In the 10 patients with non-surgical indication, there was predominance of behavioral changes (5 patients). Two had indication of drug treatment and 3 had indication of AIO use.

Among the 7 patients with positive Müller maneuver only in the oropharynx/hypopharynx, we found 2 snoring, 2 mild, 1 moderate and 2 severe cases of OSAHS. As to BMI, 3 were obese and 4 were non-obese. Four patients had PAS < 11 and 3 had PAS ^3^ 11. In these patients, surgery was indicated in 6 cases and 5 were UPFP.

## CONCLUSION

1. The most indicated initial treatment was surgery.
2. In snoring cases, surgical and non-surgical indications were at the same proportion.
3. The indication of surgical treatment was prevalent in OSAHS, regardless of the modality.
4. The surgical treatment most frequently employed was UPFP.
5. There was predominance of surgeries that interfered directly over pharyngeal soft tissues (UPFP, LAUP, uvulectomy, tonsillectomy).
6. The non-surgical treatments most frequently indicated were behavioral changes and AIO.
7. CPAP indication was restricted to patients that could be submitted to surgical treatment either temporarily or definitely.
8. BMI did not influence the modality of treatment.
9. Cephalometric analysis did not influence the option for surgical or non-surgical treatment.
10. Upon comparing modality of ventilation disorder, PAS < 11 and BMI, none of these variables had a preponderant role in the selection of surgical or non-surgical procedure.
11. The main option for surgical treatment, when comparing the ventilation disorder modality, PAS ^3^ 11 and BMI, shows importance of tonsil volume in the genesis of the obstructive process and the role of the paradoxical increase of the posterior space in the cephalometric analysis.
12. Müller maneuver did not directly interfere in the option for surgical treatment, even when it is negative in both anatomical sites studied.
13. The therapeutic decision should result from the systematic analysis of the cases and multidisciplinary approach, requiring individual analysis of each case.
